# Quantitative assessments of pupillary light reflexes in hospital-onset unresponsiveness

**DOI:** 10.1186/s12883-021-02275-9

**Published:** 2021-06-24

**Authors:** Hyunjo Lee, Soh Hyun Choi, Bobin Park, Yoon-Hee Hong, Han-Bin Lee, Sang-Beom Jeon

**Affiliations:** Department of Neurology, Asan Medical Center, University of Ulsan College of Medicine, 88, Olympic-ro 43-gil, Songpa-gu, Seoul, 05505 Republic of Korea

**Keywords:** Pupillary light reflex, Pupillometry, Prediction, Prognostication

## Abstract

**Background:**

Patients who develop hospital-onset unresponsiveness should be promptly managed in order to avoid clinical deterioration. Pupillary examination through pupillary light reflex is the gold standard method in the initial evaluation of unresponsive patients. However, the current method of shining light and subjective description often shows poor reliability. The objective of this study is to explore whether a quantitative measurement of pupillary light reflexes is useful in detecting brain herniation syndrome and predicting neurological outcomes in patients who developed hospital-onset unresponsiveness after admission for non-neurological reasons.

**Methods:**

This was a registry-based observational study on patients who activated the neurological rapid response team at Asan Medical Center (Seoul, Korea). Hospital-onset unresponsiveness was defined as a newly developed unresponsive state as assessed by the ACDU (Alert, Confused, Drowsy, and Unresponsive) scale during the hospital stay. Demographics, comorbidities, pupillometry parameters including Neurological Pupil index, brain herniation syndrome, in-hospital mortality, and modified Rankin Scale at 3-months were analyzed.

**Results:**

In 214 consecutive patients with hospital-onset unresponsiveness, 37 (17%) had brain herniation syndrome. The optimal cut-off value of Neurological Pupil index for detecting brain herniation syndrome was < 1.6 (specificity, 91% [95% confidence interval (CI) = 86–95]; sensitivity, 49% [95% CI = 32–66]). The in-hospital mortality rate was 28% (59/214); the Neurological Pupil index was negatively associated with in-hospital mortality after adjustments for the presence of brain herniation syndrome (adjusted odds ratio = 0.77, 95% CI = 0.62–0.96). Poor neurological outcomes (modified Rankin Scale ≥4) at 3 months was observed in 76% (152/201) of the patients; the Neurological Pupil index was negatively associated with poor neurological outcomes after adjustments for clinical variables (adjusted odds ratio = 0.67, 95% CI = 0.49–0.90).

**Conclusions:**

Quantitative measurements of pupillary light reflexes may be useful for early detection of potentially life-threatening neurological conditions in patients with hospital-onset unresponsiveness.

**Supplementary Information:**

The online version contains supplementary material available at 10.1186/s12883-021-02275-9.

## Background

Unresponsiveness is a challenging complaint in patients during emergency department visits and hospitalizations [1]. Various causes underlie unresponsiveness, including metabolic encephalopathy, structural brain lesions, meningoencephalitis, and seizure disorders. Early management of the underlying causes, as well as the clinical symptoms, should be promptly provided in order to avoid clinical deterioration, morbidity, and mortality [2]. Hospitalized patients generally have more comorbidities and are sicker than the general population; hence, the etiologies and outcomes of sudden unresponsiveness may differ between in-hospital patients and outpatients. Nevertheless, comprehensive studies on the early evaluations and long-term outcomes of hospital-onset unresponsiveness (HOU) are lacking.

Pupillary examinations are the gold standard in the initial evaluation of unresponsive patients [3, 4]. The assessments of pupils include diameter, shape, symmetry, and light reflexes. Evaluation of the pupillary light reflexes (PLR) is conventionally performed by shining light into the patient’s eye and subjectively described by examiners. However, such conventional assessments of PLR have low inter-rater and intra-rater correlations [5]. Recently, quantitative pupillometry (QP) was introduced to the neurocritical care field to provide objective assessments of the PLR. QP is able to detect subtle early changes in the size and light reflexes of pupils [6]. This bedside tool is becoming popular in the routine evaluation of unresponsive patients who are admitted to intensive care units and require serial measurements of pupils, especially for those with brain herniation syndrome (BHS) [7–10]. Moreover, QP was shown to be useful in neurological prognostication in unresponsive patients after cardiac arrest [11–17]. Because the PLR is regulated by the autonomic nervous system, QP may also have a role in the assessment of autonomic dysfunction in critically ill patients [18, 19]. However, studies on the QP findings in patients who are hospitalized outside intensive care units are lacking. Moreover, the clinical implication of the findings of sluggish pupils remains largely uninvestigated in unresponsive patients, although fixed dilated pupils inarguably suggest urgent situations and grave prognosis [3].

The aims of the study were the following: (1) to describe the etiology of HOU, (2) to clarify whether quantitative assessments of the PLR through QP measurement can be used to detect BHS, which is the serious in-hospital neurological complications requiring emergent interventions, and (3) to explore which QP parameters are related to clinical outcomes in patients who developed HOU after admission for non-neurological reasons.

## Methods

### Patients

This registry-based longitudinal observational study was performed between September 1, 2017 and November 30, 2018 at Asan Medical Center, a tertiary hospital located in Seoul, South Korea. All data for the current study have been collected and documented on the registry of the Neurological Alert Team (NAT), a round-the-clock neurologist-led rapid response team organized for the improvement of hospital-wide performance in response to in-patient neurological emergency [20]. For this study, we included patients who were (1) 18 years of age or older, (2) admitted to non-neurological departments, and (3) activated the NAT due to hospital-onset unresponsive state (i.e., stupor or comatose state) as assessed by the ACDU (Alert, Confused, Drowsy, and Unresponsive) scale, which is a simple four-point scale for assessing patients presenting with altered mental status [21]. The NAT neurologists or clinical nurse specialists routinely measured the patients’ pupils by using QP for initial neurological assessment during office hours. We excluded patients with no available QP measurements, incomplete demographic data, or unresponsiveness following cardiac arrest. This study was approved by the institutional review board of Asan Medical Center and the need for written informed consent was waived considering the retrospective nature of the study.

### Clinical assessments

According to our protocol, the neurologists performed initial neurological assessments as soon as possible following NAT activation. Systematic evaluations, including vital signs, ACDU scale, Glasgow Coma Scale (GCS), laboratory tests, computed tomography (CT) and/or magnetic resonance imaging (MRI) of the brain, and electroencephalography (EEG) were conducted. Basal functional status before the admission was also assessed with the modified Rankin Scale (mRS) by the neurologists, based on the information from the next of kin. Clinical outcomes were assessed by the survival status at hospital discharge and the mRS at 3 months after the day of NAT activation. The mRS at 3 months was evaluated via clinical visits, telephone interviews, and medical record review and was documented on the dedicated NAT registry [20]. Neurological status was categorized into good (mRS = 0–3) and poor (mRS = 4–6) [22]. All data were prospectively documented in our registry and were reviewed and adjudicated by the investigators at weekly conferences.

### Pupillometry measurements

QP was measured at bedside using the NPi-100 pupillometer (NeurOptics, Irvine, CA, USA). The detection thresholds were 10.00 mm for the maximum pupil diameter (Max), 1.00 mm for the minimum pupil diameter (Min), and 0.03 mm for the change in size. The QP parameters included Max (mm), Min (mm), percentage of change (CH = 100 × [Max – Min]/Max), constriction velocity (CV, mm/sec), latency of constriction (Lat, msec), dilation velocity (DV, mm/sec), and Neurological Pupil index (NPi). NPi is a scale with values ranging from 0.0 to 5.0, a combination of z-scores of Max, Min, CH, CV, Lat, and DV measurements compared against the mean distribution of values obtained from healthy volunteers [8]. Low NPi value can be interpreted as less distinct PLR (i.e., decreased pupillary reactivity). All QP assessments were performed at least twice in each eye. Among the repetitive QP measurements, the dataset with the maximum NPi value content was documented for each eye to minimize type I error, in which normal PLR is considered sluggish. From the results measured on each eye, we selected the more inferior parameters (lower NPi, lower CH, lower CV, lower DV, higher Max, higher Min, and higher Lat) for either eye for the final analysis to detect any abnormalities of PLR of either eye sensitively. As a result, each QP parameter used in the final analysis may come from different eyes.

### Etiology of hospital-onset unresponsiveness

The etiology of HOU was discussed by the neurologists at a weekly conference. The causes of HOU categorized as follows: (1) metabolic encephalopathy (e.g., sepsis, organ dysfunction, electrolyte imbalance, and drug side effects); (2) ischemic stroke; (3) hemorrhagic stroke; (4) intracranial bleeding (subdural or epidural); (5) brain tumor (primary or metastatic); (6) meningoencephalitis; (7) seizure-related events (convulsive or non-convulsive seizure, status epilepticus, or postictal status); (8) hypoxic-ischemic encephalopathy; and (9) other causes (e.g. syncope, pseudocoma, and cerebral concussion). In case an HOU had multiple causes, their contributions to the HOU were ranked. All data were prospectively documented in our registry and were reviewed and adjudicated by the investigators at weekly conferences.

The presence of BHS was radiographically and clinically evaluated. We defined BHS as the development of acute brain lesions with mass effect that were relevant to the unresponsiveness, including midline shift, brainstem compression, hydrocephalus, and diffuse cerebral edema [23]. All CT scans and MRI sequences were jointly interpreted by two investigators who were blinded to the QP data and clinical outcomes. A third investigator was consulted in cases of disagreement.

### Statistical analyses

Data are summarized as mean ± standard deviation for normally distributed continuous variables, medians and interquartile ranges for non-normally distributed continuous variables, and frequencies with percentages for categorical variables. Univariable analyses were carried out to identify the relationship between each variable and BHS or clinical outcomes; categorical variables were compared with Pearson’s χ^2^ test or Fisher’s exact test, and continuous variables were compared with the Student’s *t*-test. Variables with *p* values < 0.1 in the univariable analysis were included as candidate variables in the multivariable logistic regression model and removed by backward stepwise selection. We further performed all analyses using a forward selection procedure to confirm the final model. All QP variables significantly associated with BHS or clinical outcomes in the univariable analysis were individually added to the final clinical models to generate the odds ratio (OR) and the 95% confidence intervals (CIs) thereof. To analyze the usefulness of QP for the prediction of BHS, we calculated the predictive accuracy by sensitivity, specificity, negative predictive value, and positive predictive value. Two-tailed *p* values < 0.05 were considered statistically significant. All statistical analyses were performed using R version 3.6.2 (R Foundation for Statistical Computing, Vienna, Austria) and GraphPad Prism, version 8.3.1 (GraphPad Software, La Jolla, CA, USA).

## Results

### Patient characteristics

A total of 1442 patients activated the NAT during the study period. We included 404 (28%) patients who were in an unresponsive state as assessed by the ACDU scale at the time of on-call consultations to the NAT; and excluded 190 patients who either did not have QP measurements due to NAT activation during after-hours (*n* = 129), showed unresponsiveness following cardiac arrest (*n* = 46), or had incomplete demographic data (*n* = 15). Thus, the remaining 214 patients were included in the final analysis; their mean age was 63.9 ± 14.9 years, and 127 (59%) were men. The baseline characteristics of the 214 patients are presented in Table 1.

**Table 1:** Baseline Characteristics

Factor	***n*** = 214
Age, yr, mean ± standard deviation	63.9 ± 14.9
Male sex, n (%)	127 (59.3)
Modified Rankin Scale before admission (interquartile ranges)	2 (0–4)
Modified Rankin Scale before admission ≥4, n (%)	72 (33.6)
**Comorbidities**	
Hypertension, n (%)	101 (47.2)
Diabetes mellitus, n (%)	69 (32.2)
Cardiac disease, n (%)	83 (38.8)
Chronic lung disease, n (%)	21 (9.8)
Chronic liver disease, n (%)	35 (16.4)
Chronic kidney disease, n (%)	41 (19.2)
Cancer, n (%)	85 (39.7)
Previous stroke, n (%)	34 (15.9)
**Hospital departments**	
Cardiothoracic surgery, n (%)	28 (13.0)
Gastroenterology, n (%)	22 (10.2)
Liver transplantation surgery, n (%)	22 (10.2)
Oncology, n (%)	20 (9.3)
Pulmonology, n (%)	20 (9.3)
Cardiology, n (%)	18 (8.4)
Hematology, n (%)	15 (7.0)
Acute care surgery, n (%)	13 (6.0)
Obstetrics and gynecology, n (%)	8 (3.7)
Rehabilitation medicine, n (%)	8 (3.7)
Nephrology, n (%)	7 (3.2)
Infection, n (%)	6 (2.8)
Acute emergency care unit, n (%)	5 (2.3)
Neurosurgery, n (%)	5 (2.3)
Kidney transplantation, n (%)	4 (1.8)
Orthopedic surgery, n (%)	4 (1.8)
Others, n (%)^a^	9 (4.2)
**Elapsed time to NAT activation**	
From hospital admission to NAT activation, day (interquartile ranges)	7.5 (2.0–22.8)
From last-known-normal to NAT activation, hours (interquartile ranges)	3.0 (0.5–11.9)
From first-found-abnormal to NAT activation, minutes (interquartile ranges)	53.5 (12.2–230.8)
**Findings on NAT activation**	
Glasgow Coma Scale, median (interquartile ranges)	6 (3–8)
Hypotension, n (%)	16 (7.5)
Tachycardia, n (%)	125 (58.4)

*NAT* Neurological Alert Team

^a^ Geriatric internal medicine (n = 2), Urology (n = 2), Endocrinology (n = 1), Vascular surgery (*n* = 1), Rheumatology (n = 1), Endocrine surgery (n = 1), and Plastic surgery (n = 1)

### Etiology of hospital-onset unresponsiveness

**Fig. 1:**
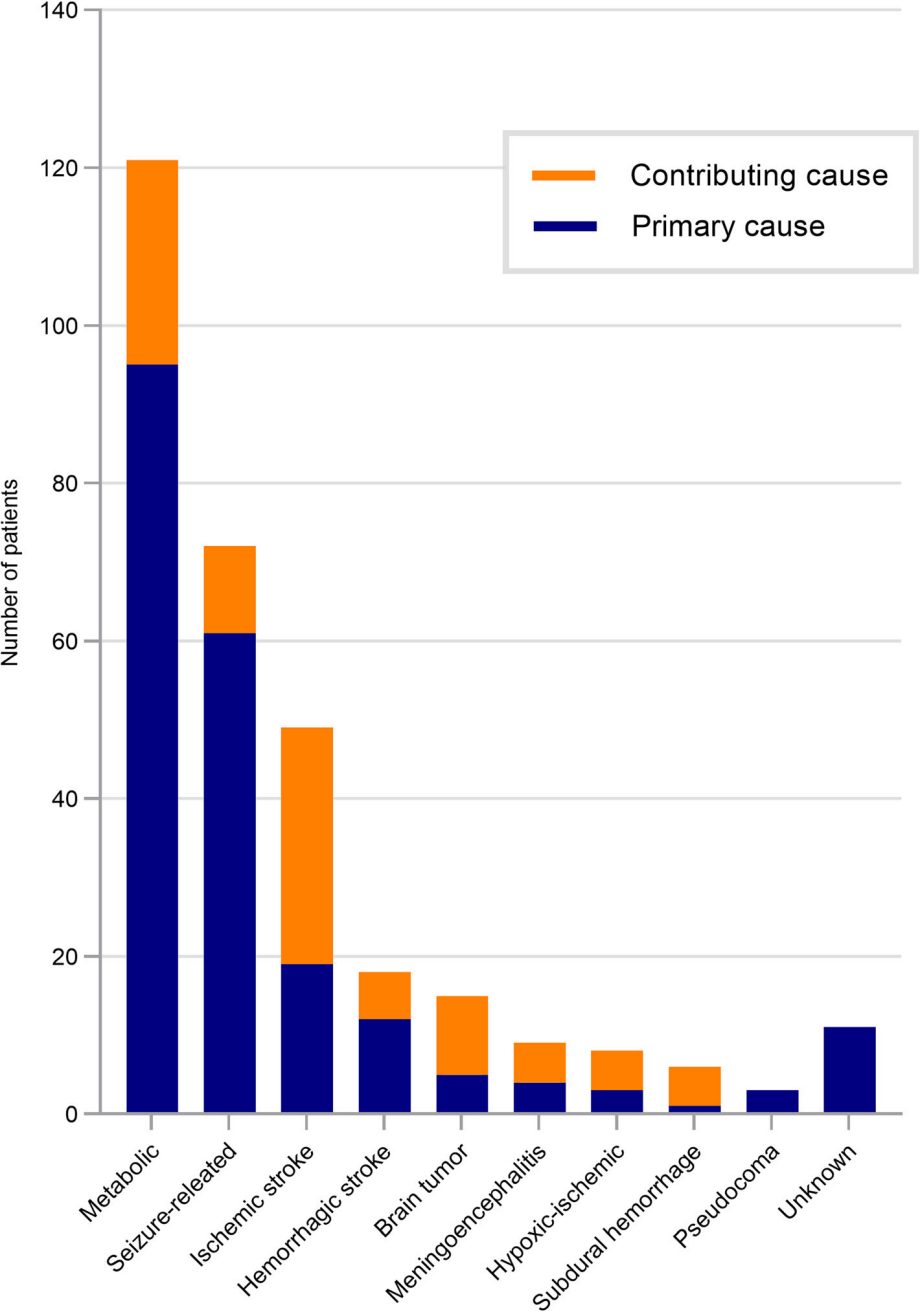
Etiologies of hospital-onset unresponsiveness. Etiologies were categorized into primary and contributing causes in 214 patients who were admitted to the hospital for non-neurological diseases

Of the included patients, 195 (91%) underwent CT and/or MRI of the brain, which showed acute intracranial lesions in 104 (53%) patients. Of them, 91 patients had brain lesions relevant to the HOU, and 13 patients had brain lesions that were irrelevant to the HOU (acute small infarcts). EEG was performed in 174 (81%) patients, including continuous EEG monitoring in 25 patients. The identified causes of HOU in 214 patients are shown in Fig. 1. The most common cause of HOU was metabolic encephalopathy (95/214), followed by seizure-related events (61/214) and ischemic stroke (19/214).

### Brain herniation syndrome

BHS was found in 37 (17%) patients, who showed higher in-hospital mortality rate compared with those without BHS (57% [21/37] vs. 21% [38/177]; *p* <  0.001). The diagnoses of the patients with BHS were hemorrhagic stroke (*n* = 10), ischemic stroke (*n* = 6), acute liver failure (n = 6), brain tumor or leptomeningeal seeding with acute hydrocephalus (n = 6), hypoxic-ischemic brain injury (*n* = 3), meningoencephalitis (*n* = 2), subdural hemorrhage (n = 2), cerebral venous thrombosis (*n* = 1), and unknown etiology of diffuse cerebral edema (n = 1). In univariable analysis, younger age, good functional status before admission, absence of a history of hypertension or diabetics, and lower GCS score at NAT activation were significantly associated with the risk of BHS (Table 2, Additional File 1: Table S1). Among the QP parameters, lower NPi, larger Max, and larger Min showed independent association with the risk of BHS on multivariable analysis. The NPi cut-off value of < 1.6 provided the maximum area under the receiver operating characteristic (ROC) curve (0.750) for predicting BHS, with a sensitivity of 49% (95% CI = 32–66) and a specificity of 91% (95% CI = 86–95) (Additional File 1: Figure S1). In addition, the positive predictive value and negative predictive value of this NPi cut-off value for predicting BHS were 53% (95% CI = 39–67) and 89% (95% CI = 86–92), respectively.

**Table 2:** Factors Associated with the Presence of Brain Herniation Syndrome

Factor	Univariable analysis OR (95% CI)	***p*** value	Multivariable analysis Adjusted OR (95% CI)	***p*** value
Age, yr	0.95 (0.93–0.97)	< 0.001	0.97 (0.94–0.99)	0.014
Male sex	1.00 (0.49–2.10)	0.987		
Modified Rankin Scale before admission ≥4	0.32 (0.11–0.77)	0.017		
**Comorbidities**				
Hypertension	0.40 (0.18–0.85)	0.021		
Diabetes mellitus	0.27 (0.09–0.68)	0.011		
Cardiac disease	0.71 (0.32–1.49)	0.384		
Chronic lung disease	0.47 (0.07–1.74)	0.331		
Chronic liver disease	2.25 (0.94–5.12)	0.058		
Chronic kidney disease	0.32 (0.07–0.96)	0.072		
Cancer	1.04 (0.49–2.13)	0.911		
Previous stroke	0.59 (0.16–1.63)	0.357		
**Findings on NAT activation**				
Glasgow Coma Scale	0.79 (0.67–0.93)	0.005	0.83 (0.71–0.98)	0.027
Hypotension	1.61 (0.43–4.97)	0.431		
Tachycardia	1.78 (0.83–4.10)	0.151		
**Quantitative measures on pupillometry**				
NPi	0.54 (0.42–0.68)	< 0.001	0.57 (0.45–0.74)	< 0.001
Max, mm	1.75 (1.32–2.37)	< 0.001	1.54 (1.14–2.09)	< 0.001
Min, mm	1.74 (1.23–2.54)	0.002	1.51 (1.03–2.21)	0.034
CH, %	0.96 (0.92–1.00)	0.066		
CV, mm/sec	0.76 (0.46–1.20)	0.273		
Lat, msec	1.00 (0.99–1.00)	0.227		
DV, mm/sec	0.42 (0.14–1.16)	0.111		

*CH* percentage of change (CH = 100 × [Max – Min]/Max), *CI* confidence interval, *CV* constriction velocity, *DV* dilation velocity, *Lat* latency of constriction, *Max* maximal pupillary diameter, *Min* minimal pupillary diameter, *NAT* Neurological Alert Team, *NPi* Neurological Pupil index, *OR* odds ratio

### In-hospital mortality

In-hospital mortality was noted in 59 (28%) patients. The presence of hemorrhagic stroke (12% [7/59] vs. 3.2% [5/155], *p* = 0.034) and presence of BHS (36% [21/59] vs. 10% [16/155], *p* <  0.001) were significantly associated with in-hospital mortality. NPi was significantly lower in patients with in-hospital mortality than in those without (3.2 [1.2–4.2] vs. 4.0 [3.2–4.4], *p* = 0.001). The following QP variables were also significantly associated with in-hospital mortality: smaller CH (14.5 ± 9.3 vs. 20.0 ± 10.5%, *p* = 0.001), slower CV (1.0 ± 0.8 vs. 1.4 ± 0.9 mm/sec, *p* = 0.004), longer Lat (331.8 ± 69.0 vs. 297.4 ± 51.9 msec, *p* = 0.002), and slower DV (0.4 ± 0.4 vs. 0.6 ± 0.4 mm/sec, *p* = 0.005). In multivariable analysis, the presence of BHS (OR = 4.80, 95% CI = 2.28–10.09, *p* <  0.001), NPi (OR = 0.77, 95% CI = 0.62–0.96, *p* = 0.019), CH (OR = 0.95, 95% CI = 0.92–0.98, *p* = 0.004), CV (OR = 0.56, 95% CI = 0.36–0.86, *p* = 0.008), Lat (OR = 1.01, 95% CI = 1.00–1.02, *p* = 0.001), and DV (OR = 0.32, 95% CI = 0.13–0.78, *p* = 0.011) were independently associated with in-hospital mortality.

### Neurological outcomes

**Fig. 2:**
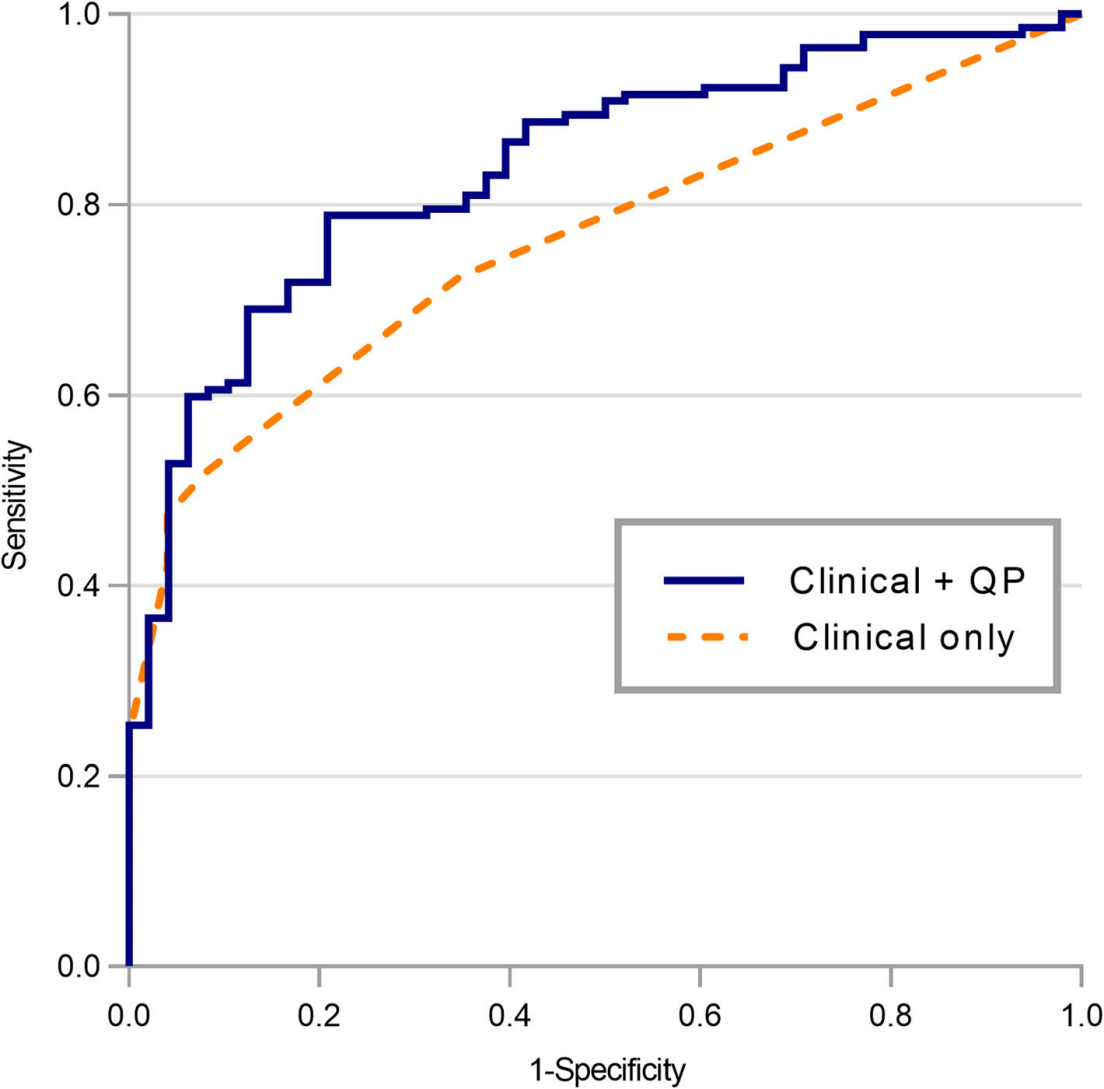
Receiver operator characteristic curves showing accuracy for the prediction of poor neurological outcomes at 3 months. The AUC for clinical variables (pre-admission modified Rankin Scale ≥4, cancer, and previous stroke) were 0.762 (a dash line); and the AUC for QP parameters (Neurological Pupil index, percentage of change, latency of constriction, and dilation velocity) in addition to aforementioned clinical variables were 0.837 (a green line). *AUC* area under the curve, *QP* quantitative pupillometry

A total of 201 (94%) patients had available data on mRS at 3 months. The median mRS at 3 months was 5 (interquartile ranges 3–5). Poor neurological outcome was noted in 152 (76%) patients. Table 3 and Additional File 1: Table S2 show the factors associated with poor neurological outcomes in patients with HOU, which included age, pre-admission mRS ≥ 4, diabetes mellitus, cancer, and previous stroke. The following QP Parameters were also associated with poor neurological outcomes at 3 months: NPi, CH, CV, Lat, and DV. Multivariable analysis showed that pre-admission mRS ≥ 4, cancer, previous stroke, NPi, CH, Lat, and DV were significantly associated with poor neurological outcomes at 3 months. The area under the ROC curve for poor neurological outcomes at 3 months was 0.762 [95% CI = 0.694–0.830] in the model with clinical variables (pre-admission mRS ≥ 4, cancer, and previous stroke). Although not statistically significant, the area under the ROC curve for poor neurological outcomes at 3 months was increased to 0.837 [95% CI = 0.784–0.894] upon addition of the QP variables (NPi, CH, Lat, and DV) (Fig. 2). The optimal cut-off value of NPi for the poor neurological outcomes at 3 months was 3.0, with sensitivity of 33% (95% CI = 26–41), specificity of 82% (95% CI = 68–91), positive predictive value of 85% (95% CI = 75–91), and negative predictive value of 28% (95% CI = 25–32).

**Table 3:** Factors Associated with Poor Neurological Outcomes at 3-months

Factor	Univariable analysis OR (95% CI)	***p*** *value*	Multivariable analysis Adjusted OR (95% CI)	***p*** *value*
Age, yr	1.03 (1.01–1.06)	< 0.001		
Male sex	1.47 (0.76–2.82)	0.241		
Modified Rankin Scale before admission ≥4	17.09 (5.03–107.0)	< 0.001	13.7 (3.14–60.6)	< 0.001
**Comorbidities**				
Hypertension	0.86 (0.45–1.65)	0.661		
Diabetes mellitus	2.66 (1.25–6.22)	0.015		
Cardiac disease	1.00 (0.51–1.98)	0.989		
Chronic lung disease	0.96 (0.35–3.09)	0.945		
Chronic liver disease	1.00 (0.43–2.54)	0.984		
Chronic kidney disease	1.91 (0.79–5.35)	0.176		
Cancer	2.24 (1.12–4.69)	0.025	2.24 (1.00–5.00)	0.031
Previous stroke	5.30 (1.51–33.6)	0.026	4.89 (1.05–22.7)	0.043
**Findings on NAT activation**				
Glasgow Coma Scale	0.89 (0.77–1.01)	0.091		
Hypotension	1.17 (0.34–5.37)	0.809		
Tachycardia	1.01 (0.50–2.00)	0.960		
**Etiology of hospital-onset unresponsiveness**				
Metabolic encephalopathy	1.07 (0.56–2.08)	0.817		
Ischemic stroke	2.76 (0.74–17.9)	0.185		
Hemorrhagic stroke	3.74 (0.70–69.3)	0.211		
Intracranial bleeding (subdural or epidural)	1.29 (0.18–25.7)	0.817		
Brain tumor	3.69 (0.20–67.9)	0.448		
Meningoencephalitis	2.32 (0.12–45.6)	0.754		
Seizure	0.56 (0.28–1.12)	0.098		
Hypoxic-ischemic encephalopathy	2.32 (0.12–45.6)	0.754		
Others (syncope or psychogenic coma)	0.06 (0.00–1.32)	0.094		
**Brain herniation syndrome**	1.69 (0.69–4.75)	0.276		
**Quantitative measures on pupillometry**				
NPi	0.72 (0.54–0.93)	0.020	0.67 (0.49–0.90)	0.009
Max, mm	0.95 (0.74–1.21)	0.695		
Min, mm	1.08 (0.78–1.54)	0.619		
CH, %	0.95 (0.92–0.98)	0.008	0.96 (0.93–1.00)	0.046
CV, mm/sec	0.57 (0.39–0.83)	0.003		
Lat, msec	1.01 (1.00–1.02)	< 0.001	1.01 (1.00–1.02)	0.002
DV, mm/sec	0.27 (0.12–0.60)	0.001	0.34 (0.14–0.84)	0.019

Neurological outcomes were dichotomized into good outcome (mRS of 0–3) and poor outcome (mRS of 4–6)

*CH* percentage of change (CH = 100 × [Max – Min]/Max), *CI* confidence interval, *CV* constriction velocity, *DV* dilation velocity, *Lat* latency of constriction, *Max* maximal pupillary diameter, *Min* minimal pupillary diameter, *mRS* modified Rankin Scale, *NAT* Neurological Alert Team, *NPi* Neurological Pupil index, *OR* odds ratio

## Discussion

In this registry-based observational study, we showed that the application of QP was useful for detecting BHS and predicting clinical outcomes in patients with HOU. When the patients’ altered mental status activated the neurological rapid response team, the initial detection of reduced pupillary reactivity (lower values of NPi) as well as enlarged pupillary size (Max and Min) were significantly associated with later identifications of BHS. The specificity and negative predictive value of the finding of NPi < 1.6 were as high as ~ 90% in predicting the presence of BHS, while the sensitivity and positive predictive value were relatively low at ~ 50%. A previous study examined the predictive values of PLR using a conventional penlight method in patients presenting to an emergency room in comatose states (GCS score less than eight) [4]. The absence of manual PLR in at least one eye showed a high sensitivity of 83% (95% CI = 76–90) but a relatively low specificity of 77% (95% CI = 69–85) for predicting structural causes of coma. In addition, the sensitivity of anisocoria more than 1 mm in this population was only 39% (95% CI = 30–48), while the specificity was at 96% (95% CI = 79–92) [4]. QP parameters indicating reduced pupillary reactivity were significantly associated with in-hospital mortality (lower values of NPi, CH, CV, and DV, and higher values of Lat) and poor neurological outcome at 3 months (lower values of NPi, CH, and DV, and higher values of Lat). The addition of QP in considering the patients’ medical conditions enhanced the prediction value (larger area under the ROC curve) of mRS at 3 months. To the best of our knowledge, this is the first study to show the prognostic value of measuring QP in patients who are admitted for non-neurological illness and develop sudden unresponsiveness.

In terms of the etiologies of HOU, we found that metabolic encephalopathy was the most common cause, followed by seizure-related events, ischemic and hemorrhagic strokes, and metastatic and primary brain tumors. Approximately 43% of our study population had acute diseases of the brain such as stroke, intracranial bleeding, brain tumor, meningoencephalitis, and hypoxic-ischemic brain injury. BHS, one of the most severe life-threatening neurologic emergencies, was found in 17% of patients with HOU and was caused by massive cerebral infarcts, subdural hemorrhage, or diffuse brain swelling related to metabolic conditions (eg, acute liver failure). Seizure-related events including convulsive seizure, nonconvulsive status epilepticus, and postictal state were also as frequent as 29%.

We found that the use of QP may be useful for the early detection of BHS. The pupillary examination is a crucial component in the initial evaluation of unresponsive patients, but early signs of pupils relevant to BHS can be nonspecific or elusive despite careful examination. In emergency situations, it is challenging to quickly perform and properly interpret neurological examinations, especially in hospitalized patients with multiple comorbidities. According to the experience of our neurological rapid response team, QP is a practical tool when used in addition to routine neurological examination, because it is portable and can be quickly performed at bedside. In a previous preliminary observational study, midline shift and increased intracranial pressure were associated with a decrease of CV below 0.6 mm/sec; however, patients with diffuse brain edema and without midline distortion did not show such a decrease in CV until the intracranial pressure exceeded 30 mmHg [10]. In our study, 13 of 37 patients with BHS had diffuse brain swelling but did not have midline shifts, which may account for the absence of differences in CV between the BHS and non-BHS groups. Nevertheless, we demonstrated that the NPi value of less than 1.6 has a high specificity and a high negative predictive value for detecting BHS in patients with HOU. Collectively, these findings suggest that patients who develop unresponsiveness during the hospital stay and show decreased NPi value on QP measurements would benefit from undergoing expeditious neuroimaging studies to detect BHS.

Traditionally, the PLR has been rather subjectively assessed by using a variety of non-standardized light sources by practitioners with varying levels of skills in neurological examinations. Recent studies have shown that such subjective assessments of pupillary reactivity have subpar reliability, with low inter-rater and intra-rater correlations [5]. Recently, the QP has become popular as it provides a non-invasive, hand-held implementation of neuro-monitoring. QP also offers an objective and standardized measurement of the PLR, and mean QP values in healthy volunteers (Max 3.5–5.3 mm, Min 2.4–3.4 mm, CH 29–36%, Lat 0.22–0.27 s, CV 1.5–2.9 mm/sec, and DV 0.9–2.2 mm/sec) have been suggested in prior studies [7, 10, 24–26]. Additionally, the automated algorithm of QP provides an NPi value derived from a combination of QP variables. An NPi score below 3.0 is generally considered an abnormal finding of sluggish light reflex [8]. Preliminary studies conducted in intensive care units reported the usefulness of QP for detecting a wide range of conditions including increased intracranial pressure [7–10], response to osmotherapy [27], discrimination between compressive lesions and microvascular ischemic oculomotor nerve palsy [28], assessment of disease severity of aneurysmal subarachnoid hemorrhage [29], the depth of sedation and analgesia [30], and neurological prognostication in traumatic brain injury and comatose resuscitation-of-spontaneous-circulation following cardiac arrest [11–17, 31]. The prognostic implication of QP has been investigated in patients with cardiac arrest [11–17], in whom a decrease in NPi values less than 2.0 was associated with unfavorable neurological outcomes [13]. Our results showed that the QP was useful in detecting BHS and predicting clinical outcomes in patients with HOU, and that decreased NPi values and increased pupillary sizes were significantly related to BHS. Decreased NPi values were also associated with in-hospital mortality and unfavorable neurological outcomes at 3-months. In terms of QP variables, the pupillary size (Max and Min) was not significantly related to mortality rate or neurological outcomes, whereas other QP variables such as CH, Lat, and DV were associated with clinical outcomes including in-hospital mortality and neurological status at 3-months. Therefore, a combination of QP variables may be more reliable than the NPi alone in the prediction of neurological outcomes, despite the high predictive value of the NPi. In summary, our results show that measurements of PLR with QP may be useful in the early detection of life-threatening neurological conditions and neuro-prognostication for patients with HOU.

Fixed or dilated pupils are generally ominous neurological signs, as sluggish or absent PLR may indicate the compression or stretching of the dorsal midbrain in which the Edinger-Westphal nuclei is located, or of the efferent oculomotor nerve that carries parasympathetic fibers [32]. Some studies suggested that the integrity and function of PLR may also be affected by the perfusion defect to the brainstem or alterations of neurotransmitter release [33, 34]. Furthermore, even though pupillary constriction by light stimulus is predominantly integrated by the parasympathetic nervous system, it is possible that sympathetic activities are also engaged in the regulation of the pupillary reactivity. First, the sympathetic nerves are involved in the dilation phase of PLR: the supranuclear inhibition via sympathetic neurons suppresses the pre-ganglionic parasympathetic neurons at the Edinger-Westphal nucleus, resulting in relaxation of the pupil sphincter muscle; also, the sympathetic neurons contract the iris dilator muscle via peripheral sympathetic innervation [32]. Second, the reticular activating system affects the pupil size and PLR by tonic inhibitory input of the Edinger-Westphal complex through releasing norepinephrine [35]. Third, cognitive and emotional processes may result in mydriatic reaction by the input of cortical innervation into the brainstem, although the exact circuits remain poorly understood [35]. Thus, the dynamics of the pupil reactivity may be indicative of lesions or dysfunctions of the cortex, subcortex, and brainstem that affect the parasympathetic system, sympathetic system, neurotransmitters, and their complex interactions.

Further studies are needed to clarify the mechanism underlying the association between QP values and clinical outcomes in patients with HOU in the absence of BHS. It is possible that autonomic dysfunction, as well as multiorgan dysfunction and brain dysfunction (e.g., metabolic encephalopathy and BHS) may underlie such association. Autonomic dysfunction mediated by inflammatory response likely has an important role in the pathogenesis of the dysfunction of the brain or other organs [36]. Neurotransmitter imbalance such as cholinergic deficiency due to inflammation and multiorgan dysfunction in critically ill patients can lead to pupillary dysfunction [18, 33]. In this context, although there is limited evidence on the association between QP values and the severity of encephalopathy [37], QP as an indicator of autonomic and brain dysfunction may play a role in assessing the severity of metabolic encephalopathy as the cause of HOU.

In addition to the inherent limitations of its single-center retrospective design, the present study has the following limitations. First, we did not assess the pupillary dilation reflex, which may be evoked by sensory stimulation and predominantly mediated by the sympathetic nervous system. Instead, we measured the DV, which could reflect sympathetic activity during the dilation phase of the PLR. Second, concurrent use of drugs that could confound the evaluation of the PLR and responsiveness such as opioids, anticholinergics, or sedative agents was not evaluated. Although a previous study showed that the use of these medications within therapeutic doses does not significantly suppress the PLR [38], we cannot exclude the possibility that other medications such as propofol may have affected the PLR [39]. Third, we did not analyze confounders such as underlying pathology of the retina or optic nerve as well as the influence of ambient lights on the PLR [40]. Fourth, we did not assess validated scales such as the Coma Recovery Scale-Revised to distinguish coma and other diagnostic entities in the field of consciousness research (e.g. unresponsive wakefulness syndrome, minimally conscious state and locked-in syndrome). However, we believe that simplified bedside assessment of the level of consciousness such as ACDU scale is more suitable for emergent situations such as in response to in-patient neurological emergencies. Lastly, we did not analyze the duration of HOU, which may have an association with outcomes.

## Conclusions

In conclusion, reduced reactivity and increased size of pupils were related to BHS in patients with HOU. Moreover, reduced pupillary reactivity was associated with in-hospital mortality and poor neurological outcomes at 3-months. These findings suggest that QP measurement may be useful for early detection of potentially life-threatening neurological conditions in patients who develop unresponsiveness after admission for non-neurological reasons.

## Supplementary Information

**Additional file 1 Table S1: Comparison between with and without Brain Herniation Syndrome. Table S2: Comparison between the Good and the Poor Functional Outcome Groups. Figure S1. Receiver operator characteristic curves showing accuracy for the prediction of brain herniation syndrome.** The AUC for NPi, Max, Min, Age, and GCS were 0.750, 0.699, 0.665, 0.710, and 0.646, respectively.

## Data Availability

The data that support the findings of this study are available from the corresponding author upon reasonable request.

## Abbreviations

ACDU: Alert, confused, drowsy, and unresponsive
BHS: Brain herniation syndrome
CH: Percentage of change
CI: Confidence interval
CT: Computed tomography
CV: Constriction velocity
DV: Dilation velocity
EEG: Electroencephalography
GCS: Glasgow Coma Scale
HOU: Hospital-onset unresponsiveness
Lat: Latency of constriction
Max: Maximal pupillary diameter
Min: Minimal pupillary diameter
MRI: Magnetic resonance imaging
mRS: modified Rankin Scale
NAT: Neurological Alert Team
NPi: Neurological Pupil index
OR: Odds ratio
PLR: Pupillary light reflexes
QP: Quantitative pupillometery
ROC: Receiver operating characteristic

## References

[1] Kanich W, Brady WJ, Huff JS, Perron AD, Holstege C, Lindbeck G, Carter CT (2002). Altered mental status: evaluation and etiology in the ED. Am J Emerg Med.

[2] Sprung CL, Peduzzi PN, Shatney CH, Schein RM, Wilson MF, Sheagren JN (1990). Impact of encephalopathy on mortality in the sepsis syndrome. The Veterans Administration Systemic Sepsis Cooperative Study Group. Crit Care Med.

[3] Clusmann H, Schaller C, Schramm J (2001). Fixed and dilated pupils after trauma, stroke, and previous intracranial surgery: management and outcome. J Neurol Neurosurg Psychiatry.

[4] Portran P, Cour M, Hernu R, de la Salle S, Argaud L (2017). Pupillary abnormalities in non-selected critically ill patients: an observational study. J Thorac Dis.

[5] Couret D, Boumaza D, Grisotto C, Triglia T, Pellegrini L, Ocquidant P, Bruder NJ, Velly LJ (2016). Reliability of standard pupillometry practice in neurocritical care: an observational, double-blinded study. Crit Care.

[6] Zhao W, Stutzman S, DaiWai O, Saju C, Wilson M, Aiyagari V (2016). Inter-device reliability of the NPi-100 pupillometer. J Clin Neurosci.

[7] Soeken TA, Alonso A, Grant A, Calvillo E, Gutierrez-Flores B, Clark J, Donoviel D, Bershad EM (2018). Quantitative pupillometry for detection of intracranial pressure changes during head-down tilt. Aerosp Med Hum Perform.

[8] Chen JW, Gombart ZJ, Rogers S, Gardiner SK, Cecil S, Bullock RM (2011). Pupillary reactivity as an early indicator of increased intracranial pressure: the introduction of the neurological pupil index. Surg Neurol Int.

[9] Chen JW, Vakil-Gilani K, Williamson KL, Cecil S (2014). Infrared pupillometry, the neurological pupil index and unilateral pupillary dilation after traumatic brain injury: implications for treatment paradigms. Springerplus..

[10] Taylor WR, Chen JW, Meltzer H, Gennarelli TA, Kelbch C, Knowlton S (2003). Quantitative pupillometry, a new technology: normative data and preliminary observations in patients with acute head injury. Technical note. J Neurosurg.

[11] Obling L, Hassager C, Illum C, Grand J, Wiberg S, Lindholm MG, Winther-Jensen M, Kondziella D, Kjaergaard J (2019). Prognostic value of automated pupillometry: an unselected cohort from a cardiac intensive care unit. Eur Heart J Acute Cardiovasc Care.

[12] Tamura T, Namiki J, Sugawara Y, Sekine K, Yo K, Kanaya T, Yokobori S, Roberts R, Abe T, Yokota H, Sasaki J (2018). Quantitative assessment of pupillary light reflex for early prediction of outcomes after out-of-hospital cardiac arrest: a multicentre prospective observational study. Resuscitation..

[13] Oddo M, Sandroni C, Citerio G, Miroz JP, Horn J, Rundgren M, Cariou A, Payen JF, Storm C, Stammet P, Taccone FS (2018). Quantitative versus standard pupillary light reflex for early prognostication in comatose cardiac arrest patients: an international prospective multicenter double-blinded study. Intensive Care Med.

[14] Elmer J (2018). Quantitative pupillometry after cardiac arrest. Resuscitation..

[15] Solari D, Rossetti AO, Carteron L, Miroz JP, Novy J, Eckert P, Oddo M (2017). Early prediction of coma recovery after cardiac arrest with blinded pupillometry. Ann Neurol.

[16] Suys T, Bouzat P, Marques-Vidal P, Sala N, Payen JF, Rossetti AO, Oddo M (2014). Automated quantitative pupillometry for the prognostication of coma after cardiac arrest. Neurocrit Care.

[17] Heimburger D, Durand M, Gaide-Chevronnay L, Dessertaine G, Moury PH, Bouzat P, Albaladejo P, Payen JF (2016). Quantitative pupillometry and transcranial Doppler measurements in patients treated with hypothermia after cardiac arrest. Resuscitation..

[18] Hasan S, Peluso L, Ferlini L, Legros B, Calabro L, Oddo M, et al. Correlation between electroencephalography and automated pupillometry in critically ill patients: a pilot study. J Neurosurg Anesthesiol. 2019;Publish Ahead of Print. 10.1097/ANA.0000000000000633. 10.1097/ANA.0000000000000633 31343506

[19] Solari D, Miroz JP, Oddo M, Vincent J-L (2018). Opening a window to the injured brain: non-invasive neuromonitoring with quantitative pupillometry. Annual update in intensive care and emergency medicine 2018.

[20] Jeon SB, Lee HB, Koo YS, Lee H, Lee JH, Park B, et al. Neurological emergencies in patients hospitalized with nonneurological illness. J Patient Saf. 2020;Publish Ahead of Print. 10.1097/PTS.0000000000000682. 10.1097/PTS.0000000000000682 32398541

[21] Gill M, Martens K, Lynch EL, Salih A, Green SM (2007). Interrater Reliability of 3 Simplified Neurologic Scales Applied to Adults Presenting to the Emergency Department With Altered Levels of Consciousness. Ann Emerg Med.

[22] van Swieten JC, Koudstaal PJ, Visser MC, Schouten HJ, van Gijn J (1988). Interobserver agreement for the assessment of handicap in stroke patients. Stroke..

[23] Hirzallah MI, Choi HA (2016). The monitoring of brain edema and intracranial hypertension. J Neurocrit Care.

[24] Boev AN, Fountas KN, Karampelas I, Boev C, Machinis TG, Feltes C, Okosun I, Dimopoulos V, Troup C (2005). Quantitative pupillometry: normative data in healthy pediatric volunteers. J Neurosurg.

[25] Theodossiadis PG, Achtsidis V, Theodoropoulou S, Tentolouris N, Komninos C, Fountas KN (2012). The effect of alpha antagonists on pupil dynamics: implications for the diagnosis of intraoperative floppy iris syndrome. Am J Ophthalmol.

[26] Wilson MH, Edsell M, Imray C, Wright A (2008). Changes in pupil dynamics at high altitude--an observational study using a handheld pupillometer. High Alt Med Biol.

[27] Ong C, Hutch M, Barra M, Kim A, Zafar S, Smirnakis S (2019). Effects of osmotic therapy on pupil reactivity: quantification using pupillometry in critically ill neurologic patients. Neurocrit Care.

[28] Kim HM, Yang HK, Hwang JM (2018). Quantitative analysis of pupillometry in isolated third nerve palsy. PLoS One.

[29] Natzeder S, Mack DJ, Maissen G, Strassle C, Keller E, Muroi C (2019). Portable infrared pupillometer in patients with subarachnoid hemorrhage: prognostic value and circadian rhythm of the neurological pupil index (NPi). J Neurosurg Anesthesiol.

[30] Rouche O, Wolak-Thierry A, Destoop Q, Milloncourt L, Floch T, Raclot P (2013). Evaluation of the depth of sedation in an intensive care unit based on the photo motor reflex variations measured by video pupillometry. Ann Intensive Care.

[31] Butt AA, Atem FD, Stutzman SE, Aiyagari V, Venkatachalam AM, Olson DM. et al, Contribution of pupillary light reflex assessment to Glasgow Coma Scale for prognostication in patients with traumatic brain injury. J Neurocrit Care. 2021. 10.18700/jnc.210001.

[32] Brazis PM, Joseph C, Biller J (2016). Localization in Clinical Neurology. Lippincott Williams & Wilkins.

[33] Ritter AM, Muizelaar JP, Barnes T, Choi S, Fatouros P, Ward J, Bullock MR (1999). Brain stem blood flow, pupillary response, and outcome in patients with severe head injuries. Neurosurgery..

[34] Reimer J, McGinley MJ, Liu Y, Rodenkirch C, Wang Q, McCormick DA (2016). Pupil fluctuations track rapid changes in adrenergic and cholinergic activity in cortex. Nat Commun.

[35] Larsen RS, Waters J (2018). Neuromodulatory correlates of pupil dilation. Front Neural Circuits.

[36] van Gool WA, van de Beek D, Eikelenboom P (2010). Systemic infection and delirium: when cytokines and acetylcholine collide. Lancet..

[37] Yang E, Kreuzer M, Hesse S, Davari P, Lee SC, Garcia PS (2018). Infrared pupillometry helps to detect and predict delirium in the post-anesthesia care unit. J Clin Monit Comput.

[38] Shirozu K, Setoguchi H, Tokuda K, Karashima Y, Ikeda M, Kubo M, Nakamura K, Hoka S (2017). The effects of anesthetic agents on pupillary function during general anesthesia using the automated infrared quantitative pupillometer. J Clin Monit Comput.

[39] Haddock JH, Mercante DE, Paccione R, Breaux JL, Jolley SE, Johnson JL, Connolly SE, deBoisblanc B (2017). Use of digital pupillometry to measure sedative response to propofol. Ochsner J.

[40] Ong C, Hutch M, Smirnakis S (2019). The effect of ambient light conditions on quantitative pupillometry. Neurocrit Care.

