# Supplementary material for: Quantitative assessments of pupillary light reflexes in hospital-onset unresponsiveness
Source: BMC Neurol. 2021 Jun 24;21:234. doi: 10.1186/s12883-021-02275-9 (PMC8223317; doi:10.1186/s12883-021-02275-9)
Supplement: Supplementary file 1 — Additional file 1 Table S1: Comparison between with and without Brain Herniation Syndrome. Table S2: Comparison between the Good and the Poor Functional Outcome Groups. Figure S1. Receiver operator characteristic curves showing accuracy for the prediction of brain herniation syndrome. The AUC for NPi, Max, Min, Age, and GCS were 0.750, 0.699, 0.665, 0.710, and 0.646, respectively. [file 12883_2021_2275_MOESM1_ESM.pdf]

Table S1: Comparison between with and without Brain Herniation Syndrome

Factor	No BHS (N=177)	BHS (N=37)	Effect size Index	Effect size
Age, yr	65.8 ± 14.1	54.7 ± 15.3	0.08*	Medium
Male sex, n (%)	105 (59.3)	22 (59.5)	0.001†	Insignificant
Modified Rankin Scale before admission ≥ 4, n (%)	66 (37.3)	6 (16.2)	0.17†	Small
**Comorbidities**				
Hypertension, n (%)	90 (50.9)	11 (29.7)	0.16†	Small
Diabetes mellitus, n (%)	64 (36.2)	5 (13.5)	0.18†	Small
Cardiac disease, n (%)	71 (40.1)	12 (32.4)	0.06†	Insignificant
Chronic lung disease, n (%)	19 (10.7)	2 (5.4)	0.07†	Insignificant
Chronic liver disease, n (%)	25 (14.1)	10 (27)	0.13†	Small
Chronic kidney disease, n (%)	38 (21.5)	3 (8.1)	0.13†	Small
Cancer, n (%)	70 (39.6)	15 (40.5)	0.01†	Insignificant
Previous stroke, n (%)	30 (17)	4 (10.8)	0.06†	Insignificant
**Findings on NAT activation**				
Glasgow Coma Scale, median (interquartile ranges)	6 [3-8]	5 [3-6]	0.04*	Small
Hypotension, n (%)	12 (6.8)	4 (10.8)	0.05†	Insignificant
Tachycardia, n (%)	99 (55.9)	26 (70.3)	0.1†	Small
**Quantitative measures on pupillometry**				
NPi	3.6 ± 1.2	2.1 ± 1.7	0.15*	Large
Max, mm	3.7 ± 1.2	4.7 ± 1.4	0.08*	Medium
Min, mm	2.9 ± 0.9	3.5 ± 1.3	0.05*	Small
CH, %	19.2 ± 10.5	15.3 ± 10.2	0.02*	Small
CV, mm/sec	1.3 ± 0.9	1.1 ± 0.8	0.01*	Small
Lat, msec	304.0 ± 55.3	318.3 ± 74.3	0.01*	Small
DV, mm/sec	0.6 ± 0.4	0.5 ± 0.4	0.01*	Small

*Eta Squared, the effect was categorized as insignificant for values ranging 0-0.01 and as small, moderate, and large for those above 0.01, 0.06, and 0.14, respectively.

†Cramer's V, the effect was categorized as insignificant for values ranging 0-0.1 and as small, moderate, and large for those above 0.1, 0.3, and 0.5, respectively.

BHS Brain Herniation Syndrome, *CH* percentage of change (CH = 100 × [Max – Min]/Max), *CV* constriction velocity, *DV* dilation velocity, *Lat* latency of constriction, *Max* maximal pupillary diameter, *Min* minimal pupillary diameter, *NAT* Neurological Alert Team, *NPi* Neurological Pupil index.

Table S2: Comparison between the Good and the Poor Functional Outcome Groups

Factor	Good outcome (N=49)	Poor outcome (N=152)	Effect size Index	Effect size
Age, yr	56.7 ± 18.7	65.9 ± 13.1	0.07*	Medium
Male sex, n (%)	26 (53.1)	95 (62.5)	0.08†	Insignificant
Modified Rankin Scale before admission ≥ 4, n (%)	2 (4.1)	64 (42.1)	0.34†	Medium
**Comorbidities**				
Hypertension, n (%)	24 (49)	69 (45.4)	0.03†	Insignificant
Diabetes mellitus, n (%)	9 (18.4)	57 (37.5)	0.17†	Small
Cardiac disease, n (%)	18 (36.7)	56 (36.8)	0.001†	Insignificant
Chronic lung disease, n (%)	5 (10.2)	15 (9.9)	0.005†	Insignificant
Chronic liver disease, n (%)	8 (16.3)	25 (16.5)	0.001†	Insignificant
Chronic kidney disease, n (%)	6 (12.2)	32 (21.1)	0.09†	Insignificant
Cancer, n (%)	13 (26.5)	68 (44.7)	0.15†	Small
Previous stroke, n (%)	2 (4.1)	28 (18.4)	0.17†	Small
**Findings on NAT activation**				
Glasgow Coma Scale, median (interquartile ranges)	6 [3-9]	6 [3-8]	0.01*	Small
Hypotension, n (%)	3 (6.5)	11 (7.6)	0.02†	Insignificant
Tachycardia, n (%)	29 (63)	92 (63.4)	0.01†	Insignificant
**Etiology of hospital-onset unresponsiveness**				
Metabolic encephalopathy	21 (42.9)	68 (44.7)	0.02†	Insignificant
Ischemic stroke	2 (4.1)	16 (10.5)	0.09†	Insignificant
Hemorrhagic stroke	1 (2)	11 (7.2)	0.09†	Insignificant
Intracranial bleeding (subdural or epidural)	1 (2)	4 (2.6)	0.02†	Insignificant
Brain tumor	0 (0)	5 (3.3)	0.09†	Insignificant
Meningoencephalitis	0 (0)	3 (2)	0.07†	Insignificant
Seizure	19 (38.8)	40 (26.3)	0.11†	Small
Hypoxic-ischemic encephalopathy	0 (0)	3 (2)	0.07†	Insignificant
Others (syncope or psychogenic coma)	2 (4.1)	3 (2)	0.17†	Small
**Brain herniation syndrome**	6 (12.2)	29 (19.1)	0.07†	Insignificant
**Quantitative measures on pupillometry**				
NPi	3.7 ± 1.2	3.1 ± 1.5	0.03*	Small
Max, mm	3.9 ± 1.4	3.9 ± 1.3	0.001*	Insignificant
Min, mm	2.89 ± 0.95	2.97 ± 1.03	0.001*	Insignificant
CH, %	22.0 ± 9.3	17.3 ± 10.5	0.04*	Small

CV, mm/sec	1.6 ± 0.9	1.2 ± 0.8	0.05*	Small
Lat, msec	280.6 ± 37.9	316.0 ± 63.5	0.07*	Medium
DV, mm/sec	0.7 ± 0.4	0.5 ± 0.4	0.06*	Medium

*Eta Squared, the effect was categorized as insignificant for values ranging 0-0.01 and as small, moderate, and large for those above 0.01, 0.06, and 0.14, respectively.

†Cramer's V, the effect was categorized as insignificant for values ranging 0-0.1 and as small, moderate, and large for those above 0.1, 0.3, and 0.5, respectively.

CH percentage of change (CH = 100 × [Max – Min]/Max), *CV* constriction velocity, *DV* dilation velocity, *Lat* latency of constriction, *Max* maximal pupillary diameter, *Min* minimal pupillary diameter, *NAT* Neurological Alert Team, *NPi* Neurological Pupil index.

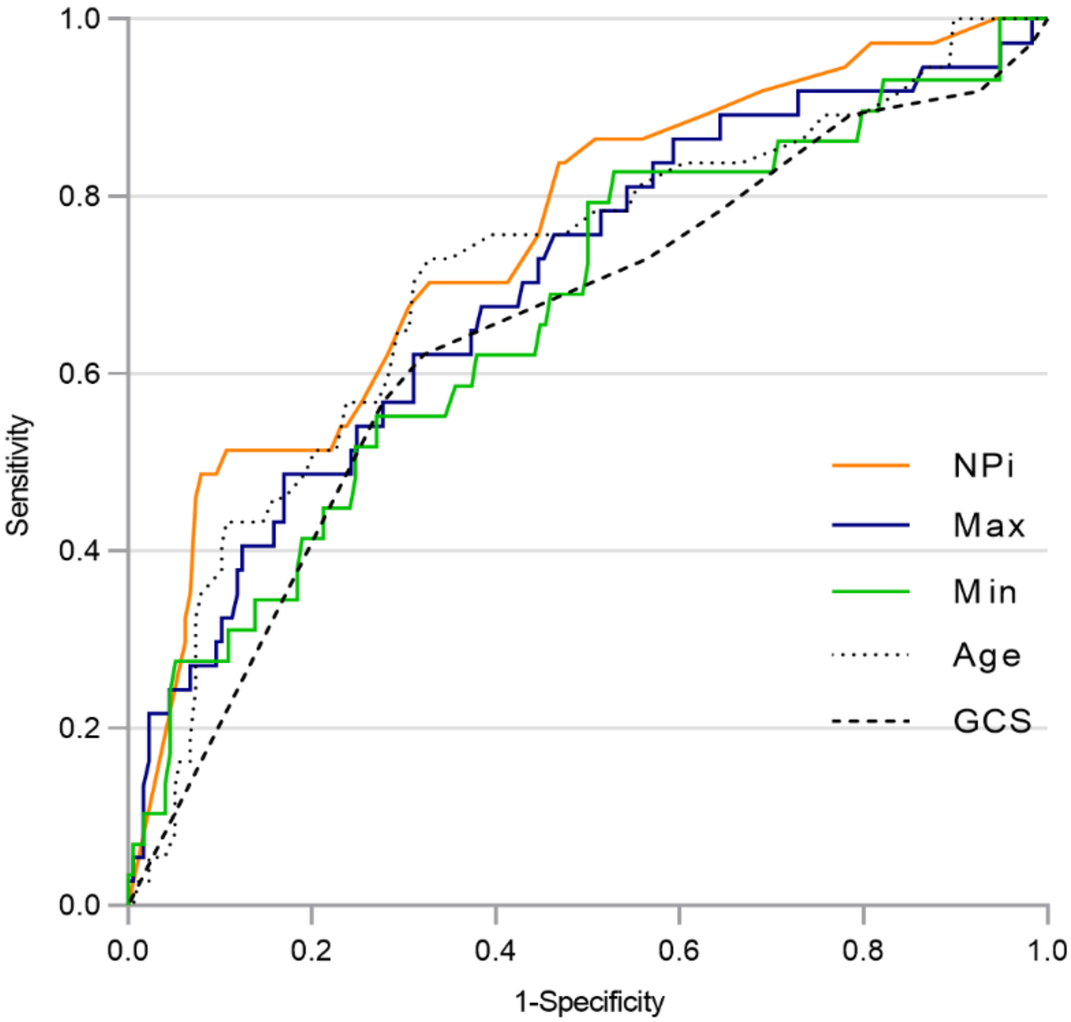

Figure S1. Receiver operator characteristic curves showing accuracy for the prediction of brain herniation syndrome. The AUC for NPi, Max, Min, Age, and GCS were 0.750, 0.699, 0.665, 0.710, and 0.646, respectively.

AUC area under the curve, *GCS* Glasgow Coma Scale, *Max* maximal pupillary diameter, *Min* minimal pupillary diameter, *NPi* Neurological Pupil index, *QP* quantitative pupillometry.
